# Glycomic Profiles of IgG, C3 and Alpha-1-Acid Glycoprotein (AGP) Before and One Year After Treatment for Active Lupus Nephritis

**DOI:** 10.3390/cells15050433

**Published:** 2026-02-28

**Authors:** Dionysis Nikolopoulos, Ana Cindrić, Konstantinos Charitidis, Natalia Sherina, Barbara Radovani Trbojević, Maja Pučić-Baković, Jelena Šimunović, Anne-Marie Patenaude, Tea Pribić, Farah Tamirou, Gordan Lauc, Frédéric A. Houssiau, Ioannis Parodis

**Affiliations:** 1 Division of Rheumatology, Department of Medicine Solna, Karolinska Institutet, Karolinska University Hospital, and Center for Molecular Medicine (CMM), SE-171 76 Stockholm, Sweden; dionysis.nikolopoulos@ki.se (D.N.); konstantinos.charitidis@ki.se (K.C.); natalia.sherina@ki.se (N.S.); 2 Genos Glycoscience Research Laboratory, Borongajska Cesta 83H, 10000 Zagreb, Croatia; acindric@genos.hr (A.C.); mpucicbakovic@genos.hr (M.P.-B.); jsimunovic@genos.hr (J.Š.); ampatenaude@genos.hr (A.-M.P.); tpribic@genos.hr (T.P.); glauc@genos.hr (G.L.); 3 Department of Rheumatology, Faculty of Medicine and Health, Örebro University, SE-701 82 Örebro, Sweden; 4 Rheumatology Department, Institut de Recherche Expérimentale et Clinique, Cliniques Universitaires Saint-Luc and Pôle de Pathologies Rhumatismales Inflammatoires et Systémiques, Université Catholique de Louvain Brussels, 1200 Brussels, Belgium; farah.tamirou@saintluc.uclouvain.be (F.T.); frederic.houssiau@uclouvain.be (F.A.H.); 5 Faculty of Pharmacy and Biochemistry, University of Zagreb, Ulica Ante Kovačića 1, 10000 Zagreb, Croatia

**Keywords:** systemic lupus erythematosus, lupus nephritis, glycomics, glycosylation, repeat kidney biopsy, outcomes

## Abstract

Background: Lupus nephritis (LN) is a severe manifestation of systemic lupus erythematosus (SLE), characterised by unpredictable outcomes due to the absence of reliable biomarkers. This hypothesis-generating study aimed to evaluate whether changes in the N-glycosylation of IgG, C3, AGP, and the serum proteins over one year of treatment correlate with clinical and histological features of LN and predict renal outcomes. Methods: Serum samples from 19 treatment-naïve patients with LN were collected at baseline and 12 months post-treatment, in conjunction with per-protocol repeat kidney biopsy. IgG (Fc, Fab, and total), C3, AGP, and total serum glycoproteins were isolated and analysed as either released N-glycans or N-glycopeptides using high-throughput glycomic approaches. Clinical and histological data were obtained at both time points, along with assessments of clinical and histological response at 12 months and long-term renal function. Results: In total, we identified 24/243 increased N-glycosylation traits (2 total IgG, 5 IgG Fc, 7 IgG Fab, 5 serum glycoproteins, 4 AGP, and 1 C3) and 10/243 decreased N-glycosylation traits (7 total IgG, 2 IgG Fc, 1 IgG Fab) following treatment. Baseline AGP IORMIF1N5H6S2F1 showed a positive correlation with eGFR both at baseline (r = 0.64, *p* = 0.005) and at 12 months (r = 0.51, *p* = 0.032). Among AGP N-glycosylation traits, IVORMI1N7H8S3 (r = 0.66, *p* = 0.002; r = 0.48, *p* = 0.041, respectively), VORMI1N8H9S4 (r = 0.51, *p* = 0.029; r = 0.49, *p* = 0.038, respectively), and VORMI1N8H9S4F1 (r = 0.48, *p* = 0.039; r = 0.49, *p* = 0.034, respectively) significantly correlated with activity index (AI) at baseline and at 12 months. Presence of cellular crescents at baseline positively correlated with three AGP N-glycosylation traits: IORMISORMIIA1N4H5S2 (r = 0.49, *p* = 0.036), VORMII1N5H6S3F1 (r = 0.63, *p* = 0.006), and VORMII1N4H5S2 (r = 0.48, *p* = 0.046). Total serum N-glycan (structure) N5H4F1 at 12 months was associated with both clinical and histological response to treatment. Delta of total serum N-glycan structure N5H5S1 was independently associated with poor long-term outcome. Conclusions: This study suggests that glycosylation changes over one year of treatment are associated with specific clinical and histological features and both short- and long-term renal outcomes in LN. Given the small cohort size, results should be considered hypothesis-generating warranting further investigation in independent cohorts.

## 1. Introduction

Lupus nephritis (LN) affects approximately 40% of patients with systemic lupus erythematosus (SLE) and remains one of its most severe and prognostically significant complications [1]. Despite advances in immunosuppressive therapies, monitoring renal disease activity and guiding treatment decisions remains challenging because reliable, non-invasive biomarkers that reflect intrarenal inflammation and chronic tissue damage are lacking [2]. Clinical and serological measures alone often fail to detect residual inflammation at the tissue level, which can persist despite an apparent clinical response, or irreversible scarring [3]. Thus, kidney biopsy remains indispensable for diagnosis, classification, and treatment guidance, while repeat biopsies can reveal important discrepancies between clinical remission and underlying histological activity [4].

Biomarkers that can predict response and both short- and long-term renal outcomes remain limited. Proteinuria levels below 0.7–0.8 g/day at 12 months from treatment initiation have been validated in multiple cohorts as a readily measurable target associated with favourable long-term prognosis [4]. However, while this target shows a high positive predictive value, its negative predictive value is poor, as many patients who fail to achieve this threshold still experience preserved renal function over time [5]. These limitations highlight the need for novel biomarkers that not only reflect histological disease activity but can also reliably predict treatment response and future renal outcomes [1].

Recent work highlights protein N-glycosylation as a critical regulator of immune homeostasis and a promising source of disease-specific biomarkers in autoimmune conditions. Alterations in IgG N-glycan structures, such as reduced galactosylation and sialylation, increased bisected GlcNAc species, and shifts in core fucosylation, have been consistently observed in SLE and associated with disease activity, serological profiles, and flares [6,7,8,9]. These glycan changes are functionally meaningful; they modulate IgG effector pathways by influencing Fcγ-receptor engagement, complement activation, and immune-complex pathogenicity, thereby contributing to a pro-inflammatory antibody phenotype [10,11]. Additional glycomic studies reinforce that IgG glycan signatures correlate with SLE activity and may capture immune dysregulation more sensitively than conventional serological markers [8,12].

Beyond IgG, broader remodelling of glycosylation in acute-phase glycoproteins and complement components has been documented in chronic inflammatory and autoimmune diseases, with increased branching and sialylation reflecting activation of systemic inflammatory pathways [13,14,15]. Collectively, these findings highlight systemic glycan signatures as a promising but underexplored class of biomarkers that may complement, refine, or even reduce reliance on invasive repeat kidney biopsies in LN.

In this study, we combined comprehensive glycomic profiling at baseline and 12 months after treatment initiation with paired histopathological data from diagnostic and repeat kidney biopsies. Our aim was to determine whether specific glycomic signatures are linked to kidney tissue-level disease activity, treatment response, and long-term renal outcome, and whether they could serve as novel biomarkers to complement, refine, or potentially reduce the need for invasive repeat kidney biopsies in managing LN.

## 2. Materials and Methods

### 2.1. Patients

This study included 19 patients fulfilling the 2019 European Alliance of Associations for Rheumatology (EULAR)/American College of Rheumatology (ACR) classification criteria for SLE [16] from the Université catholique de Louvain, who developed a biopsy-proven LN, the majority (n = 15) being incident cases (first episode of LN with no prior renal involvement), between 2017 and 2020. All study participants underwent a per-protocol repeat kidney biopsy one year (±1 month) after treatment initiation.

Serum samples were collected from each patient prior to treatment initiation (baseline) and at one year, coinciding with the diagnostic and repeat kidney biopsies. Patient rights, safety, and well-being were safeguarded in accordance with the ethical principles outlined in the Declaration of Helsinki. Written informed consent was obtained from all adult participants before enrolment. The study protocol was reviewed and approved from the Hospital-Faculty Ethics Committee of the Université catholique de Louvain and the Swedish Ethical Review Authority (reference: 2022-03907-01).

Clinical, urinary, and serological data were collected at both time points during sample collection. Kidney function was assessed based on serum creatinine (sCr) concentrations, and the corresponding estimated glomerular filtration rate (eGFR) was calculated using the 2021 CKD-EPI creatinine equation [17]. Serum albumin levels and spot urine protein-to-creatinine ratio (UPCR) were measured. Disease activity was assessed using the SLE Disease Activity Index 2000 (SLEDAI-2K) [18].

Baseline and repeat kidney biopsies were examined by light microscopy and immunofluorescence and classified according to the 2018 International Society of Nephrology/Renal Pathology Society (ISN/RPS) classification system [19]. Renal disease activity and chronic damage were assessed using the National Institutes of Health (NIH) activity index (AI) and chronicity index (CI), respectively [19]. In one patient, the repeat kidney biopsy was unsuccessful (predominantly muscle tissue was obtained). In another patient, the kidney tissue sample was insufficient to yield adequate histological data. Thus, histopathological information after treatment was unavailable for these two patients.

Clinical renal response (CRR) was defined as a UPCR lower than 0.7 g/g at month 12 following treatment initiation, in accordance with EULAR recommendations [4,5]. Histological renal response (HRR) at month 12 was defined as ≥50% reduction in the AI score, resulting in an AI score of 3 or lower, as recommended [20]. Poor long-term renal outcome was defined as a sustained sCr concentration ≥120% of the baseline value and/or a sustained sCr concentration >1.0 mg/dL at two consecutive measurements, at least three months apart, maintained through the end of the observation, as previously descripted [5,21].

### 2.2. Glycan Profiling

Glycan profiling was performed at Genos Glycoscience Research Laboratory (Zagreb, Croatia) using established high-throughput methods [22,23,24,25]. 

***Total serum protein N-glycan analysis***

For N-glycan release from total serum proteins each serum sample (10 μL) was aliquoted into 1 mL 96-well collection plates (Waters, Milford, MA, USA) followed by a deglycosylation (release of glycans) procedure. Serum proteins were denatured by the addition of 20 μL 2% (*w*/*v*) sodium dodecyl sulfate (SDS, Invitrogen, Carlsbad, CA, USA), and the sample was incubated at 65 °C for 10 min. After denaturation, 10 μL of 4% (*v*/*v*) Igepal-CA630 (Sigma-Aldrich, St. Louis, MO, USA) was added to the samples, and the mixture was shaken for 15 min on a plate shaker (GFL, Burgwedel, Germany). N-glycans were released followed by glycan release, by adding 1.2 U PNGase F (Promega, Madison, WI, USA) and incubating overnight at 37 °C.

**Fluorescent labelling and HILIC-SPE clean-up of released *N*-glycans**

Released serum N-glycans were labelled with 2-aminobenzamide (2-AB, Sigma-Aldrich, St. Louis, MO, USA). The labelling mixture consisted of 0.48 mg 2 AB and 1.12 mg 2-picoline borane (2-PB, Sigma-Aldrich) in 25 μL dimethyl sulfoxide (DMSO, Sigma-Aldrich, St. Louis, MO, USA) and glacial acetic acid (Merck, Darmstadt, Germany) (7:3, *v*/*v*) per sample. The labelling mixture was added to each sample followed by incubation at 65 °C for 2 h. Excess reagents and proteins were removed from the samples using solid phase extraction by hydrophilic interaction liquid chromatography (HILIC-SPE) using 0.2 μm wwPTFE 96-well membrane filter plates (Pall, New York, NY, USA). The N-glycans were eluted with water and stored at −20 °C until use.

**Hydrophilic interaction liquid chromatography of *N*-glycans**

2AB-Fluorescently labelled glycans were separated on a Waters Glycan Premier BEH amide chromatography column, 150 × 2.1 mm × 1.7 μm BEH particles (Waters, Milford, MA, USA), with 100 mM ammonium formate, pH 4.4 as solvent A and ACN as solvent B. A linear gradient of 30–47% ACN (Honeywell, Charlotte, NC, USA) (*v*/*v*) at a flow rate of 0.561 mL/min was used for separation in a 32.5 min analytical run. The excitation and emission wavelengths were set to 250 nm and 428 nm, respectively. The chromatograms obtained were separated into 39 peaks (GP1–GP39). Glycan peaks were assigned based on elution positions expressed in glucose units and compared with reference values in the GlycoStore database (available at: https://glycosmos.org/glycostore/uplc, accessed on 23 February 2026) for structure annotation. Glycan abundance in each peak was expressed as a percentage of the total integrated area. In addition to the 39 directly measured glycan peaks, 16 derived traits were calculated, representing summary features such as mono- and biantennary glycans; tri- and tetra-antennary glycans; glycans with 0–4 sialic acids; glycans with 0–4 galactoses; high-mannose glycans; and derived measures of branching, galactosylation, sialylation, fucosylation, and the presence of bisecting N-acetylglucosamine (GlcNAc).

**IgG glycosylation analysis**

**Isolation of IgG from human plasma**

Samples were randomly distributed across three 96-well plates. IgG was isolated from 25 μL of plasma using a CIM^®^ r-Protein G LLD 0.05 mL Monolithic 96-well plate (2 µm channels; Sartorius BIA Separations, Ljubljana, Slovenia; Cat. No. 120.1012-2) according to previously published protocols [26,27]. A vacuum-assisted setup was used, consisting of a multichannel pipette, a vacuum manifold (Pall Corporation, Port Washington, NY, USA), and a vacuum pump (Pall Corporation, Washington, NY, USA), with pressure reductions of approximately 5 mmHg during sample application and IgG elution.

Following IgG isolation, the monolithic plate was stored in 20% (*v*/*v*) ethanol in 20 mM Tris buffer containing 0.1 M NaCl (pH 7.4) at 4 °C. Subsequently, 20 μL of IgG eluate was aliquoted into a PCR plate (Thermo-Fisher Scientific, Waltham, MA, USA) and dried in a vacuum centrifuge. For IgG N-glycan analysis by capillary gel electrophoresis with laser-induced fluorescence (CGE-LIF), deglycosylation, released N-glycan labelling, and clean-up were performed using modified protocols described previously [10]. 

**N-glycan release, labeling, and clean up from IgG**

Isolated IgG samples were dried in a Savant SpeedVac vacuum concentrator and resuspended in 3 μL of 1.66× PBS (*w*/*v*). Samples were denatured by adding 4 μL of 0.5% (*w*/*v*) SDS (Sigma-Aldrich, St. Louis, MO, USA) and incubating at 65 °C for 10 min. After incubation, 2 μL of 4% Igepal CA-630 (Sigma-Aldrich, St. Louis, MO, USA) was added, followed by shaking for approximately 5 min. For N-glycan release, 1 μL of enzyme mixture containing 1.2 U PNGase F (Promega, Madison, WI, USA) in 1 μL of 5× PBS was added to each sample and incubated for 3 h at 37 °C. After incubation, samples were dried in a vacuum concentrator for 1 h, reconstituted in 2 μL of ultrapure water, and shaken for approximately 5 min prior to labelling.

The labelling mixture was freshly prepared by combining 2 μL of 30 mM 8-aminopyrene-1,3,6-trisulfonic acid trisodium salt (APTS; Synchem UG & Co., Felsberg, Germany) in 3.6 M citric acid (Sigma-Aldrich, St. Louis, MO, USA) with 2 μL of 1.2 M 2-picoline borane in dimethyl sulfoxide (Sigma-Aldrich, St. Louis, MO, USA) per sample. Samples were vortexed and incubated for 16 h at 37 °C. The labelling reaction was terminated by adding 100 μL of cold 80% acetonitrile (Carlo Erba, Cornaredo, Italy).

For clean-up, 200 μL of Bio-Gel P-10 slurry per well was added to a 0.2 μm wwPTFE AcroPrep filter plate (Pall Corporation, Port Washington, NY, USA) and used as the stationary phase. Wells were prewashed three times with 200 μL ultrapure water followed by 200 μL of 80% cold acetonitrile (*v*/*v*). Samples were loaded and incubated on a shaker for 5 min, followed by five washes with 200 μL of 80% acetonitrile containing 100 mM triethylamine (TEA; pH 8.5), and three additional washes with 80% acetonitrile. IgG N-glycans were eluted with a total of 500 μL ultrapure water and stored at −20 °C until analysis.

**IgG Fab and Fc N-Glycosylation Analysis**

Immunoglobulin G (IgG) was isolated from human plasma using a method described above. For downstream analysis, an aliquot corresponding to 50 μg of IgG was used for each sample.

Purified human IgG was enzymatically digested into Fab and Fc fragments using the IgG-specific cysteine protease IdeS. Briefly, IgG samples were first immobilised on an Fc-specific affinity matrix and washed to remove unbound material. Bound IgG was then incubated with IdeS protease in phosphate-buffered saline (pH 6.6) at 37 °C for 20 h under humidified conditions. Concentrations of the resulting Fab and Fc fractions were measured using a NanoDrop spectrophotometer to ensure consistency across samples.

Following digestion, Fab fragments were recovered in the flow-through, while Fc fragments remained bound to the affinity matrix. Fc fragments were subsequently eluted under acidic conditions and immediately neutralised to preserve labile glycan structures. Both Fab and Fc fractions were collected, dried under vacuum, and stored at −20 °C until further glycosylation analysis.

**N-glycan release, labelling, and clean-up of Fab and Fc fragments**

Fab and Fc N-glycans were released, fluorescently labelled, and purified according to established protocols for in-solution deglycosylation and APTS labelling as described above, followed by hydrophilic interaction liquid chromatography solid-phase extraction (HILIC-SPE) clean-up. All steps were performed in a high-throughput 96-well format.

**CGE-LIF Capillary gel electrophoresis analysis and data processing**

The total and Fab+Fc IgG N-glycoprofiling by capillary gel electrophoresis with laser-induced fluorescence (CGE-LIF), was performed using an Applied Biosystems 3500 Genetic Analyser (Thermo-Fischer Scientific, Waltham, MA, USA), equipped with 50 cm long 8-capillary array (Thermo-Fischer Scientific, Waltham, MA, USA). Polymer POP-7 (Thermo-Fischer Scientific, Waltham, MA, USA) was used as a separation matrix in capillaries. In a 96-well MicroAmp Optical 96-Well reaction plate, 2 μL of APTS-labeled N-glycans were combined with 8 μL of HiDi formamide, ensuring a thorough and uniform resuspension. The instrumental method was created by setting the operating parameters as follows: injection time: 9 s; injection voltage: 15 kV; run voltage: 19.5 kV; oven temperature: 60 °C; and working time: 1000 s. The obtained electropherograms were integrated in the same manner into 27 peaks using Waters Empower 3 software. The Glycan composition of each peak has been previously determined.

**Complement component 3 N-glycome measurements.**

**Lectin-based C3 enrichment.**

Complement component C3 was enriched from human plasma following the method described by Šoić et al. [23], with minor modifications. Briefly, 7.5 μL of concanavalin A (Con A)-Sepharose 4B resin was preconditioned with binding buffer (20 mM Tris-HCl, 0.5 M NaCl, pH 7.4) in a 96-well polypropylene filter plate (Orochem Technologies Inc., Naperville, IL, USA). Plasma samples (10 μL), diluted 1:9 in binding buffer, were loaded onto the conditioned resin and incubated for 2 h at 4 °C with shaking. The resin was washed three times with 250 μL of binding buffer, followed by incubation with 200 μL of elution buffer for 45 min. Eluted glycoproteins were dried in a vacuum concentrator.

**Protein digestion and glycopeptide enrichment.**

Dried samples were resuspended in 78 μL of 15% (*v*/*v*) 2-propanol in 0.1 M ammonium acetate and denatured at 60 °C for 10 min. Endoproteinase Glu-C (2 μL; 0.5 U/μL) was added, and samples were incubated overnight at 37 °C. Glycopeptides were enriched using hydrophilic interaction chromatography-based solid-phase extraction (HILIC-SPE) as previously described [23], using 85% acetonitrile instead of 90%.

**nano-LC-ESI-MS analysis.**

Enriched glycopeptides were analysed using an Ultimate 3000 RSLC nano system (Dionex, Sunnyvale, CA, USA; Thermo-Fisher Scientific, Waltham, MA, USA) coupled to an Orbitrap Exploris 240 mass spectrometer (Thermo-Fisher Scientific, Waltham, MA, USA) equipped with an EASY-Spray source. Samples (1 μL) were loaded onto an Acclaim PepMap C18 trap column (5 × 0.3 mm, 5 μm, 100 Å) at 80 μL/min and washed for 3 min. Separation was performed on an EASY-Spray PepMap RSLC C18 analytical column (75 μm × 150 mm, 3 μm particle size, 100 Å pore size) at a flow rate of 0.5 μL/min using a linear gradient. The column temperature was maintained at 35 °C.

The mass spectrometer was operated in data-dependent acquisition mode. Ionization was performed at a spray voltage of 2250 V, and full MS1 scans were acquired in the *m*/*z* range 600–2000 at a resolution of 60,000. Previously annotated glycopeptides were separated into two clusters based on peptide backbone, each containing three glycoforms. Data extraction and quantification were performed using LacyTools software [28].

**N-Glycosylation Analysis of Human Alpha-1-Acid Glycoprotein (AGP).**

**Enrichment of AGP from plasma samples.**

In each well of a 96-well PCR plate (Thermo Scientific, Waltham, MA, USA), 20 μL of plasma was mixed with 1.2 M perchloric acid (Merck) at a 1:1 (*v*/*v*) ratio and centrifuged for 20 min at 1200× g and 5 °C. The supernatant was transferred to a new PCR plate by low-speed centrifugation at 15× g for 30 s in a high-throughput manner. Subsequently, approximately 70 μL of 2% phosphotungstic acid (Sigma-Aldrich, St. Louis, MO, USA) in 2 M HCl (VWR International, Radnor, PA, USA) was added to each supernatant, followed by centrifugation for 20 min at 1200× g and 5 °C. The supernatants were discarded into an empty PCR plate using the adapter system and low-speed centrifugation. The remaining precipitate, containing the enriched AGP fraction, was solubilized by adding approximately 40 μL of 0.1 M NaOH (Sigma-Aldrich, St. Louis, MO, USA) until the solution became clear.

**Reduction, alkylation, and trypsin digestion.**

To the solubilized AGP precipitates, 5 μL of 1.5% RapiGest SF surfactant (Waters, Milford, MA, USA) in 30 mM ammonium bicarbonate (Acros Organics, Geel, Belgium) was added, and samples were incubated at 60 °C for 5 min. Subsequently, 5 μL of 60 mM dithiothreitol (DTT; Sigma-Aldrich, St. Louis, MO, USA) was added, followed by incubation for 30 min at 60 °C. After cooling to room temperature, 5 μL of 160 mM iodoacetamide (Sigma-Aldrich, St. Louis, MO, USA) was added, and samples were incubated for 30 min in the dark with shaking. Alkylation was quenched by adding 1 μL of 200 mM DTT, and pH was adjusted for trypsin digestion by adding 1 μL of 2 M ammonium bicarbonate. Tryptic digestion was performed by adding 4 μL of 0.4 μg/μL TPCK-treated trypsin (Promega, Madison, WI, USA) in 50 mM acetic acid, followed by overnight incubation at 37 °C. After digestion, 2 μL of 1 M HCl was added and samples were incubated at 37 °C for 45 min to degrade RapiGest SF.

**Glycopeptide enrichment by HILIC-SPE**

AGP glycopeptides were enriched using hydrophilic interaction liquid chromatography–based solid-phase extraction (HILIC-SPE) on a 96-well polypropylene filter plate (Orochem Technologies, Naperville, IL, USA). Five milligrams of Chromabond HILIC beads (Macherey-Nagel, Düren, Germany), prepared as a 50 mg/mL suspension in 0.1% trifluoroacetic acid (TFA) in water (Sigma-Aldrich, St. Louis, MO, USA), were added to each well. Solvent removal was performed under vacuum using a vacuum manifold (Millipore Corporation, Burlington, MA, USA). Wells were prewashed with 2 × 250 μL of 0.1% TFA in water, followed by equilibration with 2 × 250 μL of 90% acetonitrile containing 10% 0.1% TFA in water. Samples were diluted with 450 μL of 0.1% TFA in acetonitrile and loaded onto the wells. After loading, wells were washed twice with 250 μL of 90% acetonitrile containing 10% 0.1% TFA in water. Enriched glycopeptides were eluted into a PCR plate with 200 μL of 0.1% TFA in water, immediately dried in a SpeedVac vacuum concentrator (Thermo-Fisher Scientific, Waltham, MA, USA), and stored at −20 °C until analysis.

**Reversed-phase liquid chromatography–electrospray ionization MS(/MS)**

AGP glycopeptides were analysed by reversed-phase liquid chromatography coupled to electrospray ionization mass spectrometry (LC–ESI–MS/MS) using a nanoACQUITY UPLC system (Waters, Milford, MA, USA) interfaced with a Compact mass spectrometer (Bruker Daltonics, Billerica, MA, USA) via an Apollo ion source. Samples were reconstituted in ultrapure water, diluted tenfold, and loaded onto an Acclaim PepMap100 C8 trap column (5 mm × 300 μm i.d.; Thermo-Fisher Scientific, Waltham, MA, USA ), where they were washed for 3 min with 0.1% TFA in water at 40 μL/min.

Peptide separation was performed on a HALO C18 nano-LC column (150 mm × 75 μm i.d., 2.7 μm fused-core particles) using a linear gradient of solvent B (80% acetonitrile with 0.1% TFA) from 0% to 50% over 16.5 min at a flow rate of 1 μL/min and a column temperature of 30 °C. Mass spectrometry data were acquired in data-dependent mode over an *m*/*z* range of 100–4000. MS/MS spectra were collected for glycopeptide identification using stepwise fragmentation of glycan and peptide moieties. Low-abundance AGP glycopeptides were targeted using an inclusion list. Data acquisition and instrument control were performed using HyStar software (version 4.2; Bruker Daltonics, Billerica, MA, USA).

### 2.3. Statistical Analysis

Statistical analyses were conducted with R, version 4.4.2 (31 October 2024), using the tidyverse suite [29] for data pre-processing alongside purr [30] and the broom family of packages [30,31,32] for applying the statistical tests and processing the model outputs. The Wilcoxon signed-rank test was used for longitudinal paired comparisons between the N-glycosylation traits (from baseline to month 12). The Mann-Whitney U test was used for comparisons of N-glycosylation traits across clinical, histopathological, and NIH AI and CI features [19]. Multiple testing was controlled using the Benjamini-Hochberg false discovery rate (FDR) procedure, with FDR adjusted *p* values reported where adjustment was performed, denoted either as “FDR adjusted” or “*p*_value_adj” within the Supplementary Files.

For correlations between each N-glycosylation trait and clinical, NIH AI, or NIH CI scores, Spearman’s rank correlation coefficients (ρ) were calculated at baseline (baseline versus baseline), at 12 months (month 12 versus month 12), and for change over time (month 12 versus baseline). For comparisons with AI and CI, only N-glycosylation traits yielding significant differences over one year of treatment through the Wilcoxon singed-rank test were assessed, in order to reduce the multiple testing adjustment weight and limit the number of false positive associations due to the increased number of correlated glycosylation traits. Spearman’s rank correlation coefficients were also calculated for N-glycosylation trait changes in relation to clinical, AI, and CI scores at baseline, month 12, and delta values. Correlations of delta values were performed only for N-glycosylation traits that significantly changed from baseline after one year of therapy.

Logistic regression models relating N-glycosylation traits and the clinical/histopathological responses were fit univariately using Firth’s bias reduction method for baseline, month 12, and delta values, and odds ratios (ORs) with 95% confidence intervals were reported. The GLMs were performed using the logistf package [33] Univariable Cox proportional hazards models were used for time to event analyses of the N-glycosylation traits as exposures and renal impairment as the outcome, using the survival package [34]. Markers deemed significant by the model were then used in multivariable Firth penalised Cox models using the coxphf package [35]. Given the limited sample size and the high dimensionality of the glycosylation data, all association analyses were considered hypothesis-generating; correlation analyses and heatmap visualisations were considered exploratory and descriptive. Given the high dimensionality and intercorrelation of glycosylation traits, *p* values from correlation analyses were reported as nominal (unadjusted) to illustrate association patterns and generate hypotheses. These correlations were not intended to provide confirmatory evidence of statistical significance.

To reduce the risk of overfitting and small-sample bias, penalised likelihood approaches were used for both logistic (Firth’s method) and Cox regression analyses. To further limit multiple-testing burden and model instability, downstream association and regression analyses were restricted to glycosylation traits that demonstrated significant longitudinal change and/or strong univariable associations. Emphasis was placed on effect size estimates and confidence intervals in addition to *p*-values.

The ggplot2 (https://ggplot2.tidyverse.org/), ggtext (https://github.com/wilkelab/ggtext), ggrepel (https://github.com/slowkow/ggrepel), patchwork (https://patchwork.data-imaginist.com/), and grid (core R package) R packages were used for graphical visualisations, all accessed on 23 February 2026.

### 2.4. Glycosylation Notation

The glycosylation structures are reported using a monosaccharide composition shorthand in which N denotes N-acetylhexosamine, H hexose, F fucose and S sialic acid (N-acetylneuraminic acid or Neu5Ac). In case of ambiguous or multiple assignments where peak identities are not uniquely resolved, alternative compositions are reported separated by a slash “/”.

## 3. Results

### 3.1. Patient Characteristics

Nineteen patients with active, biopsy-proven LN contributed to the analytical pipeline. Those had a mean (±standard deviation, SD) age of 34.3 ± 8.6 years and a median SLE duration of 5 years (interquartile range, IQR: 11.5 years) at baseline. Most patients were female (18/19; 94.7%) and of Caucasian origin (10/19; 52.6%), followed by African (7/19; 36.8%) and Asian (2/19; 10.5%) descendants. The mean (±SD) total SLEDAI-2K score at baseline was 16.1 ± 4.7 and the mean extra-renal SLEDAI-2K score was 7.4 ± 2.8. Clinical, urinary, and serological characteristics at baseline and at 12 months after treatment initiation are summarised in Table 1.

Seventeen patients were diagnosed with proliferative class III or IV LN with or without concurrent membranous class V LN, while two patients had pure membranous class V LN at baseline. Among those initially classified with proliferative class III/IV ± V LN, three shifted to mesangial class II LN and two to pure membranous class V LN in the repeat kidney biopsy. Histological characteristics at baseline and month 12 are summarised in Table 2. The patients received initial immunosuppressive LN treatment with intravenous low-dose cyclophosphamide (n = 15), intravenous rituximab (n = 2), or oral mycophenolate mofetil (n = 2), and subsequent remission maintenance LN treatment with oral mycophenolate mofetil (n = 10), oral azathioprine (n = 8), or intravenous rituximab (n = 1). At 12 months, 12 patients (63.2%) had achieved CRR, and 10 patients (52.6%) had achieved HRR. By the end of the observation period, 14 patients (73.7%) had developed poor long-term renal outcome.

### 3.2. Differential N-Glycosylation Traits in LN over 12 Months

**Figure 1:**
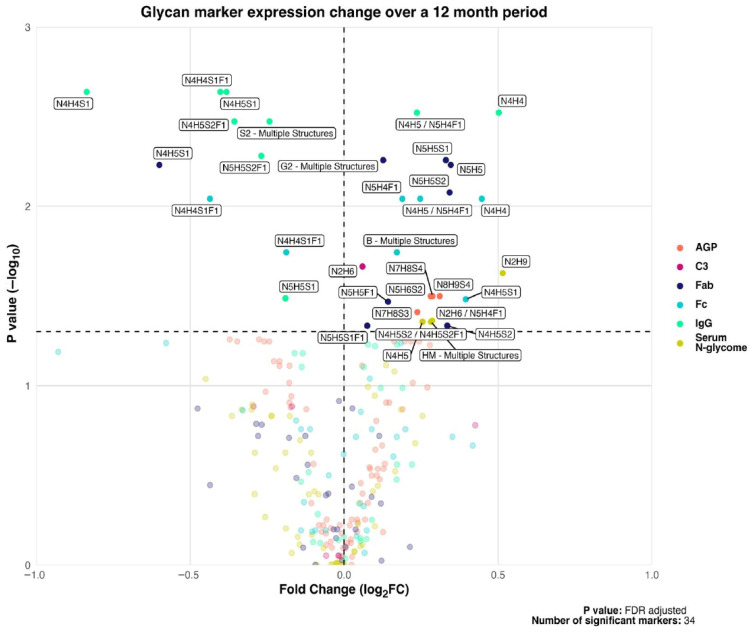
Volcano plot of glycan changes from baseline to month 12. The x axis shows log_2_ fold change (Month 12 versus Baseline); the y axis shows −log10(FDR adjusted *p* value). The horizontal dashed line marks FDR = 0.05 (−log10 = 1.3). Markers above this line are significant (opaque); non-significant markers are semi-transparent. Colours denote the protein source: AGP: coral1; C3: deeppink3; Fab: midnightblue; Fc: cyan3; IgG: mediumspringgreen; Serum N-glycome: yellow3. AGP: Alpha-1-acid glycoprotein; C3: Complement component 3; Fab: Fragment-antigen binding portion of immunoglobulin G; Fc: Fragment crystallizable region of immunoglobulin G; IgG: immunoglobulin G; FC, fold change; FDR, false discovery rate. Total significant markers: 34. Multiple testing correction was done through the Benjamini-Hochberg false discovery rate (FDR) method.

Across the 12-month treatment period, we detected significant remodeling of the circulating N-glycome. In total, 24 of 243 glycosylation traits increased (including 2 total IgG, 5 IgG Fc, 7 IgG Fab, 5 serum glycoproteins, 4 AGP, and 1 C3) whereas 10 traits decreased (7 total IgG, 2 IgG Fc, 1 IgG Fab) (Figure 1; Supplementary File S1).

Among the upregulated traits, the largest shifts were observed in total IgG, with marked increases in a galactosylated glycan structures, GP16 N4H4 (log_2_FC = 0.50; FDR = 0.003) and GP23 N5H4F1/N4H5 (log2FC = 0.23; FDR = 0.003). Conversely, the most pronounced decreases were seen in sialylated IgG structures, including GP5 N4H4S1(log2FC = −0.83; FDR = 0.002), GP7 N4H4S1F1 (log2FC = −0.40; FDR = 0.002), and GP9N4H5S1 (log2FC = −0.38; FDR = 0.002). These results indicate a systematic loss of sialylated IgG glycans over treatment period.

### 3.3. Correlations Between N-Glycosylation Traits and Clinical Indices

**Figure 2:**
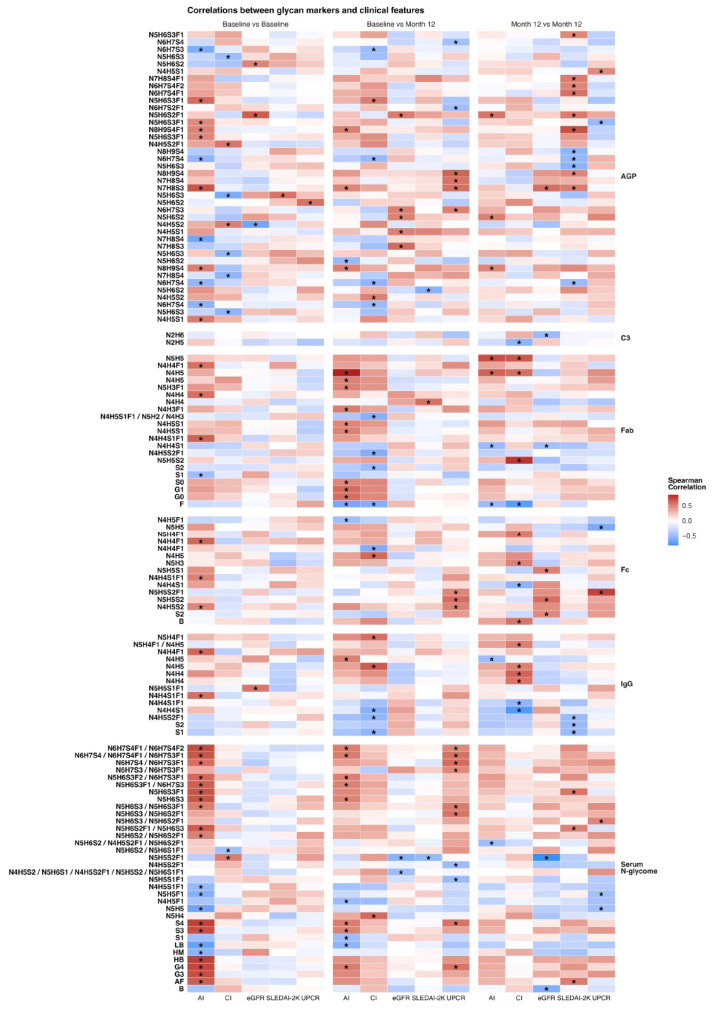
Spearman’s correlation heatmap of glycan markers with clinical indices. The heatmap demonstrates correlation values between glycan markers and clinical indices using the Spearman’s correlation metric. Correlations reaching nominal significance (unadjusted *p* < 0.05) are annotated using the asterisk symbol “*” and should be interpreted as exploratory. Columns within the heatmap denote the clinical indices, while rows denote the glycan markers (left side), grouped by origin of measurement (right side). Three types of comparisons were performed: baseline versus baseline values: month 12 versus month 12 values; baseline values versus month 12 values, as noted at the top of the heatmap. AGP: Alpha-1-acid glycoprotein; C3: Complement component 3; Fab: Fragment-antigen binding portion of immunoglobulin G; Fc: Fragment crystallizable region of immunoglobulin G; IgG: immunoglobulin G; AI: NIH renal activity index; CI: NIH chronicity index; eGFR: estimated glomerular filtration rate; SLEDAI-2k: Systemic lupus erythematosous disease activity index 2000; UPCR: urine protein to creatinine ratio.

We next investigated whether N-glycosylation traits at baseline were correlated with clinical indices at baseline and after 12 months, and whether 12-month N-glycosylation traits correlated with clinical indices at the same time point. Results are summarised on Figure 2 and in Supplementary File S2.

Several AGP glycoforms demonstrated consistent relationships with renal function and disease activity. A disialylated, fucosylated N-glycan structure at AGP site I (IORMIF1N5H6S2F1) showed a positive correlation with eGFR at baseline (r = 0.64, *p* = 0.005), which persisted at 12 months (r = 0.51, *p* = 0.032). In contrast, highly branched and highly sialylated AGP glycan structures were positively correlated with NIH Activity Index (AI) scores at both baseline and after 12 months. This was driven by the following N-glycan structure at AGP sites IV and V: IVORMI1N7H8S3 (r = 0.66, *p* = 0.002; r = 0.48, *p* = 0.041, respectively), VORMI1N8H9S4 (r = 0.51, *p* = 0.029; r = 0.49, *p* = 0.038, respectively), and VORMI1N8H9S4F1 (r = 0.48, *p* = 0.039; r = 0.49, *p* = 0.034, respectively). Beyond AGP, NIH AI scores at both baseline and 12-month follow-up showed significant correlations with nine serum N-glycans.

### 3.4. Correlations Between N-Glycosylation Traits and Specific Histopathological Features

**Figure 3:**
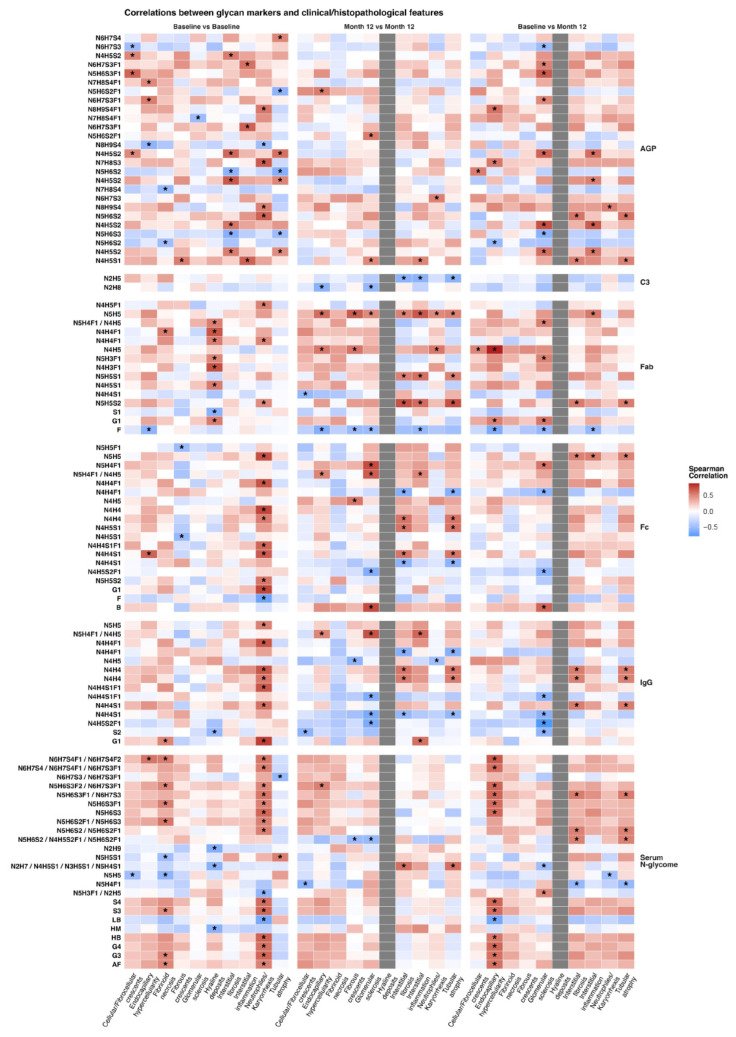
Spearman’s correlation heatmap of glycan marker values with clinical/histopathological features. The heatmap demonstrates correlation values between glycan markers and clinical/histopathological features using the Spearman’s correlation metric. Only markers who had significant differences between month 12 and baseline values were chosen for the correlation analysis. Significance was tested by performing a Mann-Whitney U test. *p* values from the Wilcoxon signed-rank test were not adjusted for false discovery rate. Correlations reaching nominal significance (unadjusted *p* < 0.05) are annotated using the asterisk symbol “*” and should be interpreted as exploratory. Columns within the heatmap denote the clinical/histopathological features, while rows denote the glycan markers (left side), grouped by origin of measurement (right side). Three types of comparisons were performed: baseline versus baseline values: month 12 versus month 12 values; baseline values versus month 12 values, as noted at the top of the heatmap. Missing values are represented with grey tiles. AGP: Alpha-1-acid glycoprotein; C3: Complement component 3; Fab: Fragment-antigen binding portion of immunoglobulin G; Fc: Fragment crystallizable region of immunoglobulin G; IgG: immunoglobulin G.

Next, we examined whether baseline N-glycosylation traits were associated with histological features at baseline and after 12 months, and whether 12-month N-glycosylation traits correlated with histological features at the same time point. Results are summarised on Figure 3 and in Supplementary File S3.

Presence of cellular crescents at baseline correlated positively with three AGP N-glycosylation traits, including sialylated bi- and tri-antennary glycan structures: IORMISORMIIA1N4H5S2 (r = 0.49, *p* = 0.036), VORMII1N5H6S3F1 (r = 0.63, *p* = 0.006), and VORMII1N4H5S2 (r = 0.48, *p* = 0.046) and negatively for one AGP N-glycosylation trait IORMISORMIIA1N6H7S3 (r = −0.49, *p* = 0.036).

Presence of wire loop lesions at baseline biopsy demonstrated strong positive correlations with -specificIgG Fab glycosylation, particularly monogalactosylated glycan structures multiple monogalactosylated N-glycan (r = 0.53, *p* = 0.021), driven by GP9 N4H5S1 (r = 0.53, *p* = 0.021), GP21 N4H4F1 (r = 0.53, *p* = 0.02), GP22 N4H4F1(r = 0.68, *p* = 0.001), and GP23 N5H4F1/N4H5 (r = 0.51, *p* = 0.027). Strong negative correlation was also demonstrated by one IgG Fab trait S1 (r = −0.58, *p* = 0.011), representing multiple monogalactosylated N-glycans. Additionally, there is positive association with agalactosylated structures such as GP15 N4H3F1 (r = 0.71, *p* = 0.0009) and GP18 N5H3F1 (r = 0.49, *p* = 0.036).

And lastly, presence of both tubular atrophy and interstitial fibrosis at baseline significantly correlated with three biantennary AGP N-glycan structures. These included a disialylated site-V structure VORMII1N4H5S2 (r = 0.58, *p* = 0.01; r = 0.61, *p* = 0.006, respectively), a trisialylated site-II structure IIORMI1N5H6S3 (r = −0.50, *p* = 0.028; r = −0.58, *p* = 0.007, respectively), a disialylated site-II structure IIORM1N4H5S2 (r = 0.47, *p* = 0.04; r = 0.54, *p* = 0.016, respectively) and two disialylated site-I structures IORMIF1N4H5S2 (r = −0.50, *p* = 0.039; r = 0.67, *p* = 0.002, respectively) and IORMIF1N5H6S2 (r = −0.59, *p* = 0.011; r = −0.56, *p* = 0.017, respectively). No significant correlations were observed between N-glycosylation traits and the presence of fibrous crescents.

### 3.5. Correlations Between Deltas of N-Glycosylation Traits and Clinical/Histological Features

**Figure 4:**
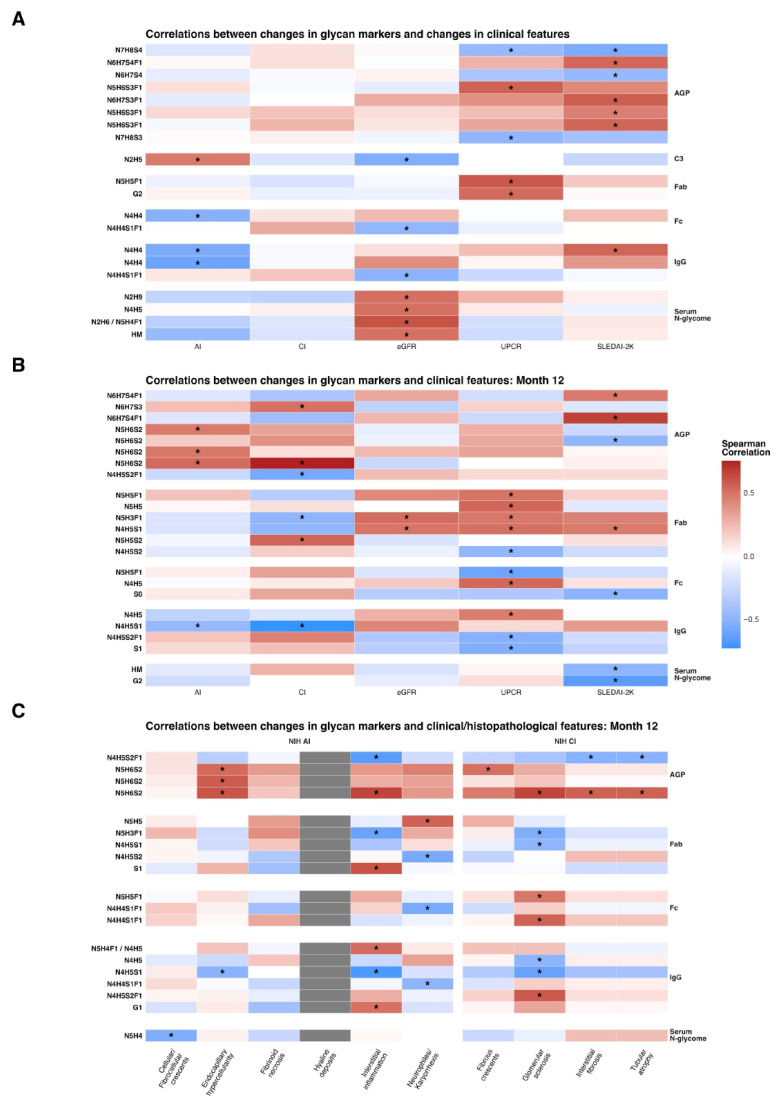
Correlations of glycan deltas with clinical and histopathological measures. (**A**) Glycan marker deltas versus baseline clinical/histopathological feature values. (**B**) Glycan marker deltas versus month 12 clinical/histopathological feature values. (**C**) Glycan marker deltas versus month 12 clinical index values. Tiles display Spearman’s correlation metrics. Only the glycan markers whose values differed significantly between month 12 and baseline were chosen for the correlation analysis. Significance was tested by performing a Mann-Whitney U test. *p* values from the Wilcoxon signed-rank test were not adjusted for false discovery rate. Correlations reaching nominal significance (unadjusted *p* < 0.05) are annotated using the asterisk symbol “*” and should be interpreted as exploratory. Columns within the heatmap denote the clinical/histopathological features, while rows denote the glycan markers (left side), grouped by origin of measurement (right side). Missing values are represented with grey tiles. AGP: Alpha-1-acid glycoprotein; C3: Complement component 3; Fab: Fragment-antigen binding portion of immunoglobulin G; Fc: Fragment crystallizable region of immunoglobulin G; IgG: immunoglobulin G.

We next evaluated whether changes in N-glycosylation traits over 12 months (calculated as 12-month minus baseline values for traits that significantly differed between time points; Figure 1) were associated with clinical (Figure 4A,B; Supplementary Files S4 and S5) and histological features (Figure 4C; Supplementary File S6) at 12 months.

While change in specific IgG monogalactosylated structure Fc GP16 N4H4 correlated negatively with changes in NIH AI scores (r = −0.52, *p* = 0.023), changes in the same glycan trait measured on total IgG, GP16 N4H4 (r = −0.6, *p* = 0.006) and GP17 N4H4 (r = −0.55, *p* = 0.014), showed negative correlation (Figure 4A).

Changes in AGP and IgG glycosylation traits showed significant correlations with NIH CI scores at 12 months. For AGP, these correlations were driven by changes in IIORM1N4H5S2F1 (r = −0.56, *p* = 0.018), IIORM1N5H6S2 N5H6S2 (r = 0.75, *p* = 0.00049), and VORMI1N6H7S3 (r = 0.5, *p* = 0.039). Among Fab-specific IgG glycans, changes in a highly sialylated structure (GP2 N5H5S2) correlated positively with NIH CI scores (r = 0.54, *p* = 0.025), whereas change in agalactosylated, bisected glycans (GP18 N5H3F1) were negatively correlated (r = –0.49, *p* = 0.047). Fab Change in total IgG GP9, a monosialylated structure (N4H5S1), also correlated negatively with CI (r = –0.72, *p* = 0.001) (Figure 4B).

At 12 months, AGP IVORMI1N5H6S2 (r = 0.5, *p* = 0.039) correlated positively with the presence of fibrous crescents, while significant correlations were also seen between IIORM1N4H5S2F1 (r = −0.50, *p* = 0.037) and IIORM1N5H6S2 (r = 0.57, *p* = 0.018) and the presence of tubular atrophy (Figure 4C).

### 3.6. N-Glycosylation Traits and Early Clinical/Histological Response to Treatment

**Figure 5:**
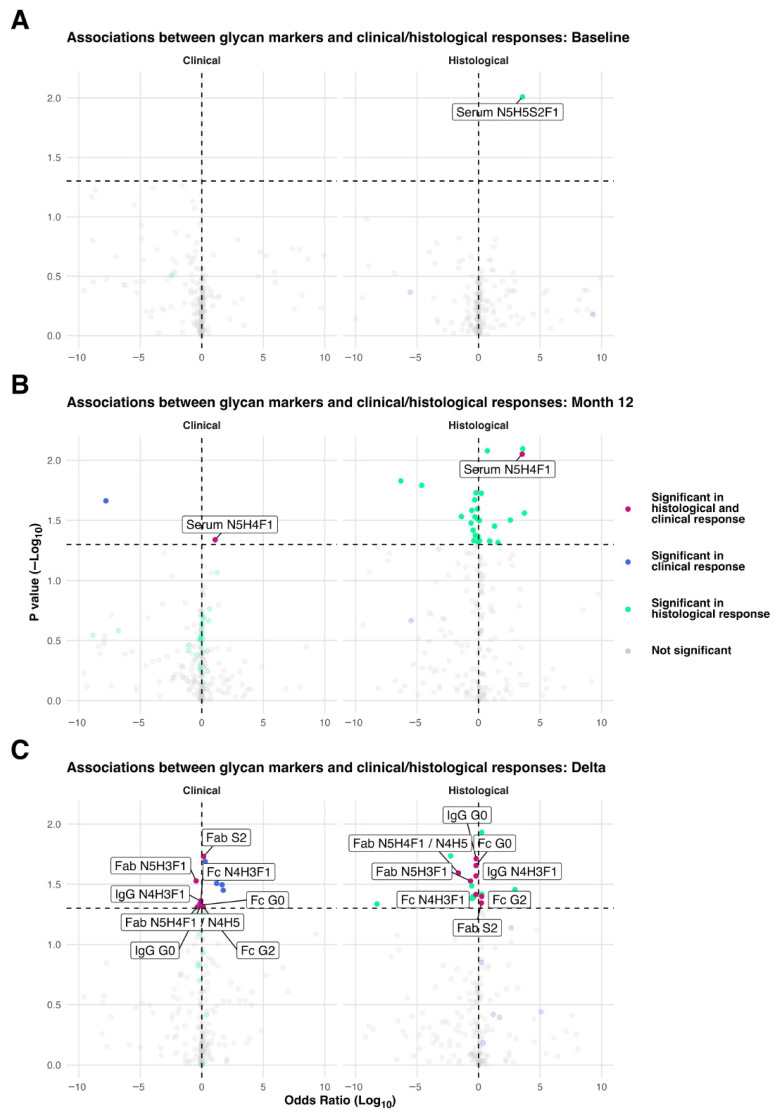
Firth-penalised logistic regression of glycan markers versus clinical and histopathological responses. (**A**) Models using glycan deltas as predictors. (**B**) Models using month 12 values. (**C**) Models using baseline values. Each point is a separate univariable model fit for one marker and one outcome. The *x*-axis shows the odds ratio (OR, log10 scale); extreme ORs (>10) are truncated. The *y*-axis shows −log10(*p* value); the horizontal dashed line marks the significance threshold (*p* = 0.05/−log10 = 1.3). Points above this line are opaque; non-significant points are semi-transparent. Colours indicate which outcome(s) reached significance: deeppink3: both clinical and histological; royalblue: clinical only; medium spring green: histological only; grey: not significant. Firth’s bias reduced logistic regression was used to address complete separation seen with standard models.

We explored whether baseline N-glycosylation traits (Figure 5A; Supplementary File S7), 12-month traits (Figure 5B; Supplementary File S8), and changes in glycan levels (Figure 5C; Supplementary File S9) were associated with early clinical and/or histological response to treatment.

Baseline levels of serum N-glycan GP23 N5H5S2F1 was associated with histological response (Figure 5A). At 12 months, 33 N-glycosylation traits (4 total IgG, 3 IgG Fab, 6 IgG Fc, 0 C3, 3 AGP, 17 serum glycoproteins) were significantly associated with histological response, whereas two traits were associated with clinical response. Serum N-glycan GP6 N5H4F1 at 12 months was significantly associated with both histological and clinical response (Figure 5B).

Changes in N-glycosylation traits showed stronger associations with both histological and clinical response compared with baseline or 12-month levels. Importantly, eight N-glycosylation traits exhibited associations with both histological and clinical response: total IgG, IgG Fc G0 (multiple agalactosylated N-glycan structures), total IgG GP15 N4H3F1, IgG Fab GP23 N5H4F1/N4H5, IgG Fab GP18 N5H3F1, IgG Fc GP15 N4H3F1, IgG Fc G2 (multiple digalactosylated N-glycan structures), and IgG Fab S2 (multiple disialylated N-glycan structures) (Figure 5C). Odds ratio, 95% confidence interval, and *p*-value are provided in Supplementary Files S8 and S9; due to the small size of the cohort, data should be interpreted with caution.

### 3.7. N-Glycosylation Traits and Long-Term Renal Outcomes

**Figure 6:**
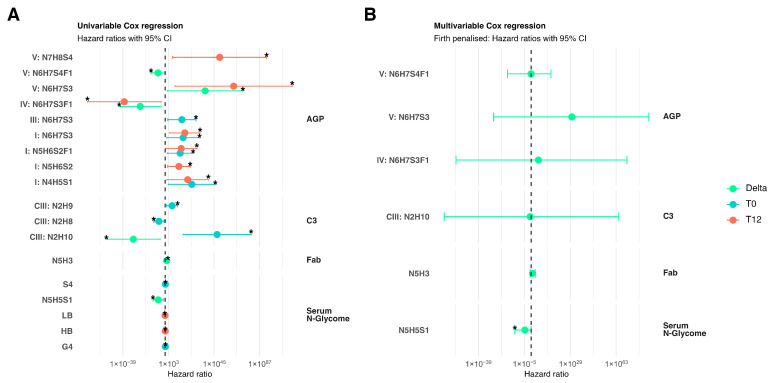
Cox regression of glycan markers and time to event long-term outcomes. (**A**) Univariable Cox regression models. Each point is the hazard ratio (HR; log scale) for one marker/value pair with its 95% CI. Colours denote which glycan value was used: Delta, T0: Baseline, or T12: Month 12. The dashed vertical line marks HR = 1 (no association). Markers with *p* < 0.05 are flagged with an asterisk; (**B**) Multivariable Firth penalised Cox models including only markers that were significant in panel (**A**) (Delta values). Points and CIs are shown as in panel (**A**). Penalisation was applied to handle separation and stabilise estimates. Groups on the right indicate the analytical origin (AGP, C3, Fab, Serum N-glycome). AGP: Alpha-1-acid glycoprotein; C3: Complement component 3; Fab: Fragment-antigen binding portion of immunoglobulin G; HR: hazard ratio.

In univariable Cox regression analysis, multiple N-glycosylation traits exhibited significant associations with long-term outcomes (Figure 6A; Supplementary File S10). Specifically, altered AGP and C3 N-glycosylation traits demonstrated the strongest associations with long-term outcomes. Both baseline and month-12 N-glycosylation traits of AGP IORMIF1N6H7S3, IORMIF1N5H6S2F1, and IORMIF1N4H5S1 were significantly associated with favourable long-term outcomes. Baseline N-glycosylation traits of C3 CIIIGIRMN1N2H10 and CIIIGIRMN1N2H9 were significantly associated with favourable long-term outcomes, while C3 was associated with poor long-term renal outcome (Figure 6A; Supplementary File S11). In multivariable Cox regression analysis, only changes in serum N-glycan GP15 N5H5S1 was independently associated with poor long-term outcomes (Figure 6B; Supplementary File S10). Odds ratio, 95% confidence interval, and *p*-value are provided in the Supplementary File S10; due to the small size of the cohort, data should be interpreted with caution.

**Figure 7:**
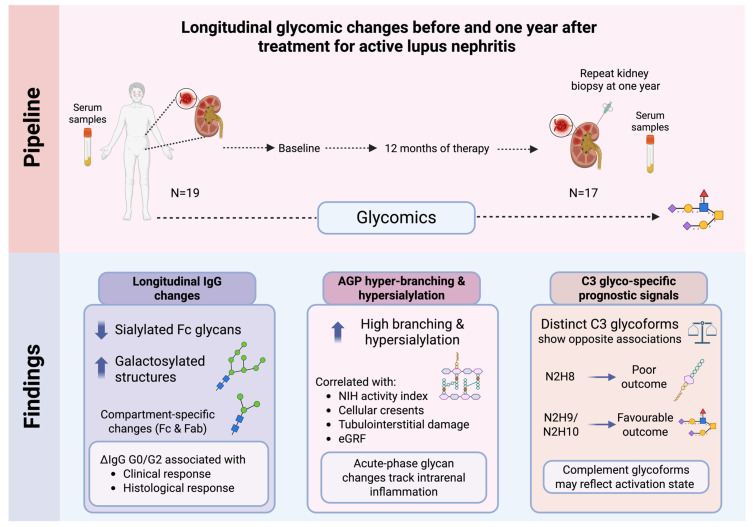
Graphical illustration of the analytical pipeline and main findings of the study. Figure was created with Biorender.

Figure 7 summarises the analytical pipeline and main findings of the study.

## 4. Discussion

In this study, we provide the first longitudinal glycomic analysis of IgG, C3, AGP, and the serum N-glycans in patients with active LN undergoing per-protocol repeat kidney biopsy, integrating alterations in protein N-glycosylation patterns with paired histopathological and clinical outcomes. We found that specific N-glycosylation traits not only changed significantly over the first year of treatment but also correlated with renal activity and chronicity indices, distinct histological features, and both clinical and histological response. Importantly, alterations in AGP and C3, as well as serum N-glycan traits, demonstrated strong associations with long-term renal outcomes. These findings point to glycomic signatures that may prove useful biomarkers in LN, with potential capacity to complement or refine current clinical and histological assessment tools.

Our findings are consistent with prior evidence showing that changes in protein glycosylation are closely linked to immune activation and inflammatory regulation in systemic autoimmune diseases [36]. Reduced sialylation of IgG has repeatedly been associated with enhanced effector function and increased inflammatory potential in rheumatoid arthritis and SLE [6,37]. A similar trend was observed in our cohort, where decreases in sialylated IgG glycans paralleled the persistence of immune activity despite apparent clinical improvement. The reduced IgG Fc sialylation observed during follow-up should be seen in relation to the established IgG glycome alterations in SLE [6]. Click or tap here to enter text.Large cohort studies have consistently shown that SLE is associated with a shift of the IgG Fc glycome towards a less anti-inflammatory configuration, including reduced terminal sialylation, and that these alterations are associated with changes in disease activity in the same direction [6,38]. Experimental data further indicate that Fc sialylation modifies IgG effector function and contributes to anti-inflammatory signalling pathways, including those implicated in the mechanism of action of intravenous immunoglobulin (IVIG) used to treat immune-mediated diseases [39,40]. Longitudinal changes in IgG sialylation during treatment are therefore likely to reflect a remodelling of the humoral immune compartment in the context of combined effects of immune activation and immunosuppressive therapy. In LN, clinical response assessment typically requires prolonged follow-up, as histopathological and immunological resolution at the tissue level may take longer time than measurable clinical improvement. Alterations in IgG Fc glycosylation may therefore occur over extended timeframes and should not be interpreted as immediate surrogates of therapeutic response, but rather as indicators of the current immune state.

Beyond IgG, the specific glycan configurations observed across C3 and AGP in our study provide mechanistic insight into how serum N-glycosylation may modulate renal inflammation and repair. While IgG is mainly produced by plasma cells [41], both complement component C3 and AGP are synthesised predominantly by hepatocytes and circulate as major acute-phase proteins. The bulk of circulating C3 is liver-derived, although limited extrahepatic production has been described under inflammatory conditions [42]. Similarly, AGP is a classical hepatocyte-produced acute-phase reactant whose expression and glycan microheterogeneity are modulated by inflammatory signalling [43]. Accordingly, glycosylation changes in AGP and C3 largely reflect systemic acute-phase pathway regulation, whereas IgG Fc glycosylation represents remodelling within adaptive immunity. Certain AGP N-glycan structures characterised by combined sialylation and fucosylation are compatible with sialyl-Lewis x motifs, which are known to interact with selectins and regulate leukocyte adhesion and recruitment under inflammatory conditions [44,45,46,47,48]. In our dataset, such glycosylation patterns, particularly one AGP form associated with favourable long-term outcomes, support the hypothesis that AGP N-glycosylation may reflect counter-regulatory or homeostatic inflammatory pathways in LN. The association between increased branching and sialylation of AGP and higher activity indices is consistent with known features of acute-phase glycoprotein remodelling. The α1-acid glycoprotein is a highly glycosylated acute-phase reactant whose N-glycan antennary and terminal sialylation undergo inflammation-associated modification [49,50]. Inflammatory signalling influences hepatic glycoprotein synthesis and glycosyltransferase activity, resulting in altered branching patterns and increased terminal sialic acid content [43,51]. Such structural changes affect the interaction properties of AGP and reflect systemic inflammatory pathway activation. Composite glycoprotein-derived inflammatory measures, including GlycA, which largely reflect circulating acute-phase proteins such as AGP, have been associated with inflammatory burden in SLE [52]. The relationship between AGP hyper-branching/hypersialylation and histological activity features in our cohort is therefore indicative of a sustained acute-phase pathway in LN, even in the setting of partial clinical improvement. Importantly, AGP-derived glycan traits primarily reflect systemic acute-phase pathway activation rather than kidney-specific processes. Similarly, alterations in IgG Fc glycosylation represent adaptive immune remodelling at a systemic level and should not be interpreted as direct surrogates of intrarenal pathology, but rather as circulating markers that may parallel intrarenal inflammatory activity. In contrast, the increase of high mannose glycans on C3, which has been linked to renal injury in diabetic nephropathy, may reflect complement activation and endothelial stress [53,54]. Together, these structural patterns suggest that specific N-glycosylation motifs across distinct serum proteins capture complementary aspects of the inflammatory and reparative balance in LN.

From a mechanistic perspective, these glycosylation changes may represent both drivers and readouts of the inflammatory milieu in active LN. Reduced galactosylation and sialylation of total IgG and IgG Fab-specific IgG glycans could facilitate enhanced immune complex formation and Fcγ receptor engagement, promoting inflammatory effector responses within glomeruli [55]. Conversely, the presence of highly sialylated and fucosylated AGP structures may act as a counter-regulatory mechanism, dampening leukocyte recruitment and reactive oxygen species–mediated injury [56]. link between a specific C3 glycoform with lower hexose content (N2H8) and poor renal outcomes further suggests that selected complement glycoforms, rather than high mannose C3 structures in general, may reflect ongoing complement activation and renal microvascular stress [23]. In contrast, closely related C3 glycoforms with higher hexose content (N2H9 and N2H10) were associated with favourable outcomes, highlighting a potential glycoform-specific relationship that warrants further exploration. Together, these results highlight that serum N-glycosylation signatures are not merely epiphenomena of systemic inflammation but may mirror and potentially influence pathogenic pathways within the kidney [55]. This integrative interpretation underscores their potential as mechanistically informed biomarkers for precision monitoring of LN.

Although this study was not designed to determine LN specificity, several of the observed glycomic patterns have been described in other immune-mediated glomerulonephritides. Compartment-specific IgG remodelling has been reported in ANCA-associated vasculitis, where Fc hypogalactosylation contrasts with comparatively more galactosylated/sialylated Fab glycans [57], while reduced IgG galactosylation and sialylation predicted relapse, supporting the concept that longitudinal glycan changes track clinical activity [37]. In primary membranous nephropathy, altered IgG4 glycosylation of anti-PLA2R1 autoantibodies promotes lectin-pathway complement activation and podocyte injury [58]. Similarly, IgA nephropathy is associated with galactose-deficient IgA1 and glycan-specific IgG autoantibodies drive pathogenic immune complex formation and associate with disease progression [59,60]. Finally, emerging data indicate that distinct C3 N-glycoforms vary with renal injury phenotypes, supporting the biological plausibility that complement glycoform heterogeneity reflects functionally distinct activation states [54]. Collectively, these comparisons suggest that our findings may reflect shared immune mechanisms across immune-mediated glomerulonephritides, whereas defining LN specificity will require direct comparisons across such diseases.

A major strength of this study lies in its longitudinal design, combining comprehensive serum glycomic profiling with paired kidney biopsies obtained before and after treatment. This unique setup allowed direct integration of molecular glycosylation changes with histological activity and chronicity indices, enabling evaluation of associations that cannot be captured through cross-sectional or clinically defined endpoints alone. The analysis of serum N-glycome and detailed profiling of multiple individual glycoproteins (IgG, C3, AGP,) represents the broadest glycomic study in LN to date and provides a broad overview of systemic and immune-related N-glycosylation changes during treatment.

However, several limitations should be acknowledged. First, the relatively small sample size; while the study design requiring paired serum samples and per-protocol repeat kidney biopsies provides unique histopathological integration, it limits statistical power and precision of effect estimates. Thus, the cohort size increases the risk of false-positive findings and model instability, particularly in regression and time-to-event analyses. Although penalised methods were applied to mitigate small-sample bias, several odds ratios and hazard ratios exhibited wide confidence intervals and should therefore be interpreted cautiously. Rather, these associations are intended to highlight candidate glycomic signals for future validation. Accordingly, the observed associations should be considered hypothesis-generating rather than definitive evidence of prognostic utility. Second, although the majority of patients (15/19) received a uniform initial treatment regimen for the induction of remission with low-dose intravenous cyclophosphamide according to the Euro-Lupus protocol, a minority received rituximab or mycophenolate mofetil. Immunosuppressive therapies, particularly B cell-depleting agents, are known to influence IgG glycosylation patterns. Thus, despite limited variability in treatment regimens, we cannot fully disentangle glycosylation changes driven by drug effects since the sample size and the small numbers of non-cyclophosphamide regimens precluded formal adjustment or subgroup analyses. Third, the exploratory nature of our study and the absence of an independent validation cohort necessitate that the observed associations should be interpreted with caution. Moreover, urinary glycomic profiling was not performed in this study. Finally, while serum N-glycosylation changes may reflect immunological or inflammatory processes occurring in the kidney, the observations of the present study cannot directly establish causality or tissue origin [38]. 

## 5. Conclusions

This study demonstrates that LN is accompanied by coordinated, longitudinal changes of protein-specific N-glycosylation patterns with distinct clinical and histopathological associations. Transition from active to treated state was associated with systematic reshaping of the IgG glycome, including a reduction in sialylated Fc structures (GP5, GP7, GP9) and enrichment of galactosylated species (GP16, GP23), with compartment-specific differences between Fc and Fab domains. In parallel, increased branching and sialylation of AGP site-specific N-glycans correlated with histological activity features such as cellular crescents, chronicity features, and renal function, indicating that acute-phase glycoprotein changes track histological inflammatory burden rather than solely systemic inflammation. Importantly, changes over 12 months, particularly shifts in IgG G0/G2 traits and serum GP15, were associated with clinical and histological response and long-term outcome to a greater extent than baseline glycomic profiles, underscoring the relevance of glycan kinetics. Furthermore, differential associations of individual C3 glycoforms (N2H8 versus N2H9/N2H10) with prognosis suggest that complement glycosylation heterogeneity may reflect distinct complement activation states.

Collectively, these findings support longitudinal glycomic profiling as a mechanistically informative biomarker layer in LN, integrating adaptive immunity, acute-phase signalling, and complement pathway activity. Moreover, our findings provide preliminary evidence that serum protein N-glycosylation patterns, particularly those of AGP, C3, and IgG, reflect histological activity and chronicity in LN and may hold prognostic value in relation to early treatment response and long-term renal outcomes. As an exploratory investigation, the present study identifies candidate glycomic signatures that warrant independent validation. Confirmation in larger, prospectively followed LN cohorts, such as the ongoing ReBioLup trial (https://rebiolup.com/) [4], will be essential to establish clinical relevance. Upon validation, glycomic signatures could complement conventional clinical and serological biomarkers and improve disease monitoring while potentially reducing reliance on repeat kidney biopsies. Future studies should aim to replicate these associations in more diverse patient populations, explore mechanistic links between specific glycan structures and immune or fibrotic pathways, and assess the temporal dynamics of glycosylation changes in relation to treatment interventions. Integration of glycomics with other omics layers, such as proteomics or transcriptomics, may further elucidate the molecular networks underlying renal inflammation and repair [61]. Overall, our results support the concept that glycosylation profiling represents a promising, minimally invasive tool, holding promise toward advancing personalised management in LN.

## Figures and Tables

**Table 1:** Clinical, urinary, and serological characteristics of LN patients at baseline and at one year.

Clinical Characteristics	Baseline (n = 19)	Repeat Kidney Biopsy at 1 Year (n = 19)
S-creatinine (mg/dL), mean (SD)	1.07 (0.56)	0.91 (0.29)
eGFR *, mean (SD), mean (SD)	78.7 (30.2)	89.6 (30.4)
Urine protein/creatinine ratio (UPCR; g/g), median (IQR)	1.65 (0.96–3.96)	0.38 (0.15–1.03)
Serum albumin (g/L), mean (SD)	29.6 (5.7)	41.9 (6.2)

* Based on the CKD-EPI creatinine equation.

**Table 2:** Histological characteristics of LN patients at baseline and at repeat kidney biopsy.

Histological Characteristics	Baseline (n = 19)	Repeat Kidney Biopsy at 1 Year (n = 17)
*ISN/RPS class*		
Class II, n (%)	0 (0)	2 (11.7)
Class III or IV, n (%)	6 (31.6)	4 (23.4)
Class III or IV + V, n (%)	11 (57.9)	6 (35.1)
Class V, n (%)	2 (10.5)	5 (29.4)
*NIH Activity Index*		
NIH activity index, mean (SD)	6 (3.2)	1.6 (1.9)
NIH activity index, median (IQR; Q1–Q3)	6 (3–8)	1 (0–3)
-Cellular/fibrocellular cresents, n (%) *	8 (44.4)	2 (11.7)
-Endocapillary hypercellularity, n (%) *	13 (72.2)	10 (58.8)
-Fibrinoid necrosis, n (%) *	8 (44.4)	1 (5.9)
-Hyaline deposits, n (%) *	7 (38.8)	0 (0)
-Interstitial inflammation, n (%) *	10 (55.5)	5 (29.4)
-Neutrophils/karyorrhexis, n (%) *	10 (55.5)	2 (11.7)
*NIH Chronocity Index*		
-NIH chronicity index, mean (SD)	2.5 (1.3)	3 (2.3)
-NIH chronicity index, median (IQR; Q1–Q3)	2 (1–2)	3 (1.5–4)
-Tubular atrophy, n (%) *	16 (88.8)	15 (88.2)
-Glomerular sclerosis, n (%) *	11 (61.1)	10 (58.8)
-Interstitial fibrosis, n (%) *	14 (77.7)	14 (82.4)

* Presence. ISN/RPS: International Society of Nephrology/Renal Pathology Society; NIH: National Institute of Health (USA).

## Data Availability

Data are available upon reasonable request.

## Supplementary Materials

The following supporting information can be downloaded at: https://www.mdpi.com/article/10.3390/cells15050433/s1, Supplementary Files S1–S11.
